# Sleep Quality and Its Determinants Among Patients with Metastatic Cancer Treated with Immune Checkpoint Inhibitors: A Two-Center Cross-Sectional Study

**DOI:** 10.3390/medicina61122131

**Published:** 2025-11-28

**Authors:** Betul Aktepe, Tugce Ulasli, Pinar Ezgi Dama, Ilkay Tugba Unek, Aziz Karaoglu, Mehmet Hamid Boztas, Suayib Yalcin, Oktay Halit Aktepe

**Affiliations:** 1 Department of Psychiatry, Urla State Hospital, Ministry of Health, Izmir 35430, Turkey; bdabakolu@gmail.com; 2 Department of Medical Oncology, Dokuz Eylul University, Izmir 35330, Turkey; 3 Department of Psychiatry, Bolu Abant Izzet Baysal University, Bolu 14030, Turkey; 4 Department of Medical Oncology, Hacettepe University Cancer Institute, Ankara 06230, Turkey

**Keywords:** immune checkpoint inhibitors, immunotherapy, Pittsburgh Sleep Quality Index, quality of life, sleep disturbance

## Abstract

*Background and Objectives*: Sleep disturbance (SD) is common among cancer patients and may be influenced by immune-related mechanisms during immune checkpoint inhibitor (ICI) therapy. This study evaluated the prevalence and predictors of SD in patients receiving ICIs. *Materials and Methods*: This retrospective, two-center study included 187 patients with advanced or metastatic cancers. Sleep quality was assessed at three months using the Pittsburgh Sleep Quality Index (PSQI). Patients were categorized as having no SD (PSQI ≤ 5) or SD (PSQI > 5). Logistic regression analyses identified predictors of SD. *Results*: Clinically relevant SD was observed in 97 patients (51.9%), with a mean PSQI score of 7.54 ± 5.39. The most affected PSQI components were SD (1.33 ± 1.05) and daytime dysfunction (1.21 ± 1.04). In univariate analyses, Eastern Cooperative Oncology Group (ECOG) performance status ≥ 1 (Odds ratio [OR]: 3.29, 95% confidence interval [CI] 1.77–6.08, *p* < 0.001), second-line or beyond therapy (OR: 3.76, 95% CI 1.92–7.34, *p* < 0.001), ≥ 2 metastatic sites (OR: 2.69, 95% CI 1.47–4.92, *p* = 0.001), and ≥6 ICI cycles (OR: 1.85, 95% CI 1.04–3.32, *p* = 0.036) were associated with SD. In multivariate analysis, ECOG ≥ 1 (OR: 2.33, 95% CI 1.17–4.62, *p* = 0.015), second-line or beyond therapy (OR: 2.43, 95% CI 1.14–5.16, *p* = 0.021), and ≥ 2 metastatic sites (OR: 2.10, 95% CI 1.06–4.16, *p* = 0.032) remained independent predictors. *Conclusions*: Over half of patients treated with ICIs experienced SD. Poor performance status, advanced disease burden, and later-line therapy independently predicted impaired sleep, supporting the routine assessment of sleep during ICIs.

## 1. Introduction

Sleep is a fundamental component of human physiology, playing a critical role in cellular recovery, immune system regulation, and optimal psychological and cognitive performance [1]. Among cancer patients, sleep disturbances (SDs) are highly prevalent and have been linked to fatigue, cognitive decline, and poorer survival outcomes [2]. The causes of SD in oncology are multifactorial, involving tumor-related symptoms, treatment toxicities, psychological distress, and altered circadian rhythms [3]. SD has been linked to impaired immune regulation and enhanced inflammatory responses, which may in turn promote tumor progression [1,4].

Immune checkpoint inhibitors (ICIs), including agents targeting programmed cell death protein 1 (PD-1), programmed death-ligand 1 (PD-L1), and cytotoxic T-lymphocyte–associated antigen 4 (CTLA-4) pathways, have transformed the treatment landscape of several cancers [5]. Despite their survival benefits, ICIs have distinct toxicity profiles, known as immune-related adverse events (irAEs), which can involve multiple organ systems and disrupt sleep regulation [6]. Proinflammatory cytokines such as interleukin (IL)-6 and tumor necrosis factor-alpha (TNF-α) can influence immune checkpoint activity and sleep regulation, indicating a mechanistic link through shared neuroimmune and inflammatory pathways that shape tumor immunity and sleep physiology [7].

The Pittsburgh Sleep Quality Index (PSQI) is a validated and widely used tool for evaluating subjective sleep quality in cancer care [8,9]. Despite its clinical relevance, SD among patients receiving ICIs remains frequently unrecognized and inadequately managed. Emerging evidence suggests that SD may impair quality of life and influence treatment adherence, immune regulation, and possibly long-term survival outcomes [10]. Given these potential implications, our study aimed to determine the prevalence and predictors of SD in cancer patients treated with ICIs, using the PSQI as a standardized assessment tool.

## 2. Materials and Methods

### 2.1. Patients and Study Design

This cross-sectional, two-center study included patients aged 18 years or older with histologically confirmed advanced or metastatic malignancies who received ICIs at the Departments of Medical Oncology of Bolu Abant Izzet Baysal University and Dokuz Eylül University between October 2022 and October 2025. Eligible systemic therapies included agents targeting PD-1 or PD-L1, such as nivolumab, pembrolizumab, or atezolizumab, either as monotherapy or in combination with other standard systemic treatments. Only patients who completed at least three cycles of ICIs were included in the analysis, while those with incomplete clinical or PSQI data or who discontinued treatment earlier were excluded. Additionally, the study included only individuals with an Eastern Cooperative Oncology Group (ECOG) performance status of 0–2. To minimize potential confounding factors, patients with primary sleep disorders unrelated to malignancy or anticancer therapy (such as obstructive sleep apnea, restless legs syndrome, or narcolepsy) and those with uncontrolled psychiatric or neurological disorders that could impair sleep quality (including severe depression, bipolar disorder, or epilepsy) were not considered eligible.

### 2.2. Sleep Quality Assessment

Sleep quality was evaluated using the PSQI, a widely validated self-report tool that assesses seven domains of sleep over the preceding month. The PSQI consists of seven component scores, including subjective sleep quality, sleep latency, sleep duration, habitual sleep efficiency, SDs, use of sleep medication, and daytime dysfunction. Each component is scored from 0 (no difficulty) to 3 (severe difficulty). The scores are summed to obtain a global score between 0 and 21, with values > 5 considered indicative of clinically significant SD. Therefore, we used this threshold to define clinically relevant SD in this study.

### 2.3. Statistical Analysis

Descriptive statistics summarized baseline characteristics. Continuous variables were expressed as mean ± standard deviation (Sd) or median with interquartile range (IQR); categorical variables as frequencies and percentages. Logistic regression models were used to identify predictors of SD. Variables with *p* ≤ 0.20 in univariate analyses were entered into multivariable models. Odds ratios (OR) with 95% confidence intervals (CI) were reported. A two-sided *p*-value < 0.05 was considered statistically significant. All analyses were performed using the Statistical Package for the Social Sciences, Version 27.0 (IBM Corp., Armonk, NY, USA).

## 3. Results

### 3.1. Patients’ Clinical Characteristics

A total of 187 patients who received ICIs were included in the analysis. Demographic and clinical features for both the overall cohort and the PSQI-based groups are presented in Table 1. The median age of the cohort was 60 years, with 93 patients (49.7%) aged < 60 years and 94 (50.3%) aged ≥ 60 years. The majority of participants were male (72.7%, *n* = 136), and most had an ECOG performance status of 1–2 (61%). The most common primary cancer type was non–small cell lung cancer (NSCLC) in 105 patients (56.1%), followed by renal cell carcinoma (RCC) in 27 (14.4%), melanoma in 20 (10.7%), and other malignancies in 35 (18.7%). Bone, lung, liver, and adrenal metastases were present in 43.3%, 28.3%, 20.9%, and 13.4% of patients, respectively. Hypertension (HT) was the most common comorbidity (26.2%), followed by diabetes mellitus (DM, 10.2%) and cardiovascular disease (CVD, 11.8%). Most patients (69.5%) received ICIs as second-line or later therapy, while 30.5% were treated in the first-line setting. Anti-PD-1-based regimens constituted the majority of treatments (82.9%), followed by anti-PD-L1 (13.4%) and anti-CTLA-4/combination regimens (3.7%).

The median number of ICI cycles at the time of sleep assessment was 6 (IQR: 4–9). When stratified according to sleep quality, 97 patients (51.9%) were classified as having SD (PSQI > 5), and 90 (48.1%) as having good sleep quality (PSQI ≤ 5). SD was more frequent among patients aged ≥ 60 years than those aged <60 years, although the difference was not statistically significant (*p* = 0.068). The proportion of male patients was slightly higher in the non–SD group compared with the SD group (*p* = 0.068). SD was more frequent in the ECOG 1–2 group than in the ECOG 0 group (*p* < 0.001). Primary cancer type and comorbidities did not differ significantly between groups (*p* > 0.05 for all). SD was more frequent among those receiving ICIs as second-line or beyond therapy compared with those treated in the first-line setting (*p* < 0.001). SD was significantly more frequent among patients receiving ICIs as second-line or beyond therapy and among those who had completed six or more treatment cycles at the time of PSQI assessment (*p* < 0.001 and *p* = 0.036, respectively). No significant difference was observed in the distribution of anti-PD-1, anti-PD-L1, or anti-CTLA-4/combination regimens between the groups (*p* = 0.641).

### 3.2. Sleep Quality Components and Predictors of SD

The mean PSQI component scores and predictors of SD are summarized in Table 2 and Table 3. Among the seven PSQI subdomains, SD (mean 1.33 ± 1.05) and daytime dysfunction (1.21 ± 1.04) were the most impaired, followed by sleep duration (1.19 ± 0.99). The lowest mean score was observed for the use of sleep medication (0.36 ± 0.74). The overall mean PSQI global score was 7.54 ± 5.39, indicating that the majority of patients experienced clinically relevant sleep impairment during ICI therapy.

Univariate and multivariate logistic regression analyses were conducted to identify factors associated with SD. In the univariate model, poorer performance status (ECOG ≥ 1) (OR: 3.29, 95% CI 1.77–6.08, *p* < 0.001), treatment with ICIs in second- or later-line settings (OR: 3.76, 95% CI 1.92–7.34, *p* < 0.001), having two or more metastatic sites (OR: 2.69, 95% CI 1.47–4.92, *p* = 0.001), and receiving ≥ 6 cycles of ICI (OR: 1.85, 95% CI 1.04–3.32, *p* = 0.036) were significantly associated with SD. In the multivariate model, SD was independently predicted by ECOG ≥ 1 (OR: 2.33, 95% CI 1.17–4.62, *p* = 0.015), second-line or beyond therapy (OR: 2.43, 95% CI 1.14–5.16, *p* = 0.021), and the presence of ≥ 2 metastatic sites (OR: 2.10, 95% CI 1.06–4.16, *p* = 0.032).

## 4. Discussion

In this cross-sectional study, we assessed PSQI-defined sleep quality in a cohort mainly comprising NSCLC, RCC, and melanoma patients receiving ICIs, with evaluations conducted after a median of six treatment cycles, and found that nearly 52% of patients had clinically meaningful SD, highlighting a substantial sleep burden and the need for routine assessment during ICI therapy. Our results are consistent with previous reports that sleep problems are common among cancer patients, with prevalence estimates ranging from 25 to 59% [11].

In cancer, insomnia is recognized as a common symptom contributing to poor quality of life and poor functioning [12]. Although sleep problems are common in oncology, they have been little studied in the context of ICIs [13]. For example, a recent pilot study in patients receiving first-line ICIs reported clinically significant insomnia in 58% of participants, with no link between insomnia severity and the number of ICI infusions [13]. In contrast, our study evaluated overall sleep quality using the PSQI in a cohort primarily composed of second-line or later-line ICI recipients who had previously received systemic therapies and likely accumulated greater symptom burden and treatment-related toxicities. These differences in both patient population and assessment method may account for the observed association between prolonged ICI exposure (≥ 6 cycles) and poorer sleep quality, which was not evident in the first-line pilot study. Li et al. reported in a prospective cohort of advanced NSCLC patients receiving sintilimab, camrelizumab, or tislelizumab that SD was an independent predictor of worse progression-free and overall survival, underlining the prognostic importance of early sleep assessment [10]. Furthermore, meta-analytic data by Kiss et al. showed an insomnia incidence of 8.3% in ICI-treated patients. Collectively, these findings emphasize that routine early follow-up with standardized tools, such as the PSQI, may facilitate timely supportive interventions.

Recent evidence highlights the potential role of chronomodulation in oncology. A systematic review of 18 randomized controlled trials involving 2547 patients demonstrated that chronomodulated chemotherapy reduced treatment-related toxicities in the majority of studies, while maintaining comparable antitumor efficacy and, in some cases, improving outcomes [14]. In the ICI setting, earlier administration of immunochemotherapy was significantly correlated with improved survival outcomes in NSCLC [15]. Additionally, Strøm et al. performed a longitudinal study to identify how sleep and circadian rest–activity rhythms evolve in NSCLC patients receiving ICIs, and they demonstrated that while sleep quality improved modestly during treatment, persistent circadian disruption remained and was closely associated with higher levels of fatigue, depression, and perceived stress [16]. However, we did not have the opportunity to record the exact time of ICIs’ infusions, but the high rate of SD may still be linked to circadian disruption caused by treatment schedules.

The biological mechanisms linking immune checkpoint inhibition to SD are complex and likely bidirectional. ICIs enhance T-cell activation and increase the release of proinflammatory cytokines, particularly IL-1β, IL-6, and TNF-α [17], which are established regulators of sleep–wake homeostasis and have been shown to modulate slow-wave sleep and circadian rhythm stability [18]. Elevated systemic inflammation may therefore establish a proinflammatory milieu that interferes with both sleep continuity and restorative sleep quality [19,20]. In addition, endocrine irAEs, well-recognized toxicities of ICIs, disturb hormonal homeostasis and can promote circadian misalignment, fatigue, and insomnia-like symptoms [6,21]. Treatment of such adverse effects often involves high-dose corticosteroids, which are well known to disrupt circadian cortisol rhythms and provoke insomnia [11]. Taken together, these mechanisms indicate that SD may serve as a marker of immune imbalance during ICI therapy, influencing both well-being and treatment outcomes.

The present study has several strengths. First, it represents one of the largest two-center cohorts to date evaluating sleep quality in patients receiving ICIs, thereby enhancing the generalizability of the findings. Second, sleep quality was assessed using the well-validated PSQI, which allows a comprehensive and standardized evaluation across multiple sleep domains. Third, the inclusion of key clinical factors, including ECOG performance status, treatment line, metastatic burden, and the total number of ICI cycles, enabled robust multivariate modeling to identify independent predictors of SD.

This study has several limitations. First, sleep quality was assessed only once, approximately three months after the initiation of ICI therapy, and no baseline pre-treatment sleep data were available. Therefore, it was not possible to determine whether SD was pre-existing or developed after ICI exposure, nor to evaluate temporal changes in sleep quality. As a result, the present findings should be interpreted as reflecting short-term sleep status during ongoing ICI therapy. Second, although patients with uncontrolled psychiatric or neurological disorders were excluded to reduce major confounding, milder psychological symptoms common in cancer populations may still have influenced subjective sleep reports but were not systematically assessed in this retrospective dataset. Third, other factors that could affect sleep quality, including pain severity, concomitant medications such as corticosteroids, and supportive care interventions, were not consistently documented. Finally, the cross-sectional design limits causal inference; future prospective studies with repeated assessments at multiple timepoints are needed to better characterize the evolution and long-term impact of ICIs on sleep.

## 5. Conclusions

Our results underscore the importance of proactively monitoring sleep in patients undergoing prolonged ICI therapy. Therefore, oncologists and supportive care teams should integrate standardized sleep assessments, such as the PSQI or validated insomnia questionnaires, into the routine follow-up of patients treated with ICIs. Optimizing sleep and circadian regulation may enhance the effectiveness of ICIs, contributing to improved treatment outcomes, reduced symptom burden, and better quality of life in cancer patients.

## Figures and Tables

**Table 1:** Baseline clinical and demographic characteristics of patients stratified according to SD status (PSQI-based).

Characteristics	All Patients (*n* = 187)	No SD Group (*n* = 90, 48.1%)	SD Group (*n* = 97, 51.9%)	*p* Value
Age (years)				0.068
<60	93 (49.7%)	51 (56.7%)	42 (43.3%)	
≥60	94 (50.3%)	39 (43.3%)	55 (56.7%)	
Gender				0.068
Male	136 (72.7%)	71 (78.9%)	65 (67%)	
Female	51 (27.3%)	19 (21.1%)	32 (33%)	
ECOG performance status				<0.001
0	73 (39%)	48 (53.3%)	25 (25.8%)	
1–2	114 (61%)	42 (46.7%)	72 (74.2%)	
Primary cancer type				0.818
NSCLC	105 (56.1%)	50 (55.6%)	55 (56.7%)	
RCC	27 (14.4%)	14 (15.6%)	13 (13.4%)	
Melanoma	20 (10.7%)	11 (12.2%)	9 (9.3%)	
Others	35 (18.7%)	15 (16.7%)	20 (20.6%)	
Metastatic sites				
Lung	53 (28.3%)	25 (27.8%)	28 (28.9%)	0.869
Liver	39 (20.9%)	17 (18.9%)	22 (22.7%)	0.524
Bone	81 (43.3%)	36 (40%)	45 (46.4%)	0.378
Adrenal	25 (13.4%)	11 (12.2%)	14 (14.4%)	0.657
Comorbidities				
HT	49 (26.2%)	25 (27.8%)	24 (24.7%)	0.637
DM	19 (10.2%)	11 (12.2%)	8 (8.2%)	0.369
CVD	22 (11.8%)	11 (12.2%)	11 (11.3%)	0.852
Line of ICI				<0.001
First-line	57 (30.5%)	40 (40.7%)	17 (31.2%)	
Second-line or beyond	130 (69.5%)	50 (59.3%)	80 (68.8%)	
ICI cycle at SD assessment				0.036
<6 cycles	89 (47.6%)	50 (55.6%)	39 (40.2%)	
≥6 cycles	98 (52.4%)	40 (44.4%)	58 (59.8%)	
Regimen type				0.641
Anti-PD-1	155 (82.9%)	77 (85.6%)	78 (80.4%)	
Anti-PD-L1	25 (13.4%)	10 (11.1%)	15 (15.5%)	
Anti-CTLA-4/combination	7 (3.7%)	3 (3.3%)	4 (4.1%)	

Abbreviations: CVD: cardiovascular disease; DM: diabetes mellitus; ECOG: Eastern Cooperative Oncology Group; HT: hypertension; ICI: immune checkpoint inhibitor; NSCLC: non–small cell lung cancer; PD-1: programmed cell death protein 1; PD-L1: programmed death-ligand 1; PSQI: Pittsburgh Sleep Quality Index; RCC: renal cell carcinoma; SD: sleep disturbance.

**Table 2:** Mean scores of the seven PSQI components and the global PSQI score among patients receiving ICIs.

Variable	Mean	Sd
Subjective sleep quality	1.12	0.99
Sleep latency	1.02	1.08
Sleep duration	1.19	0.99
Habitual sleep efficiency	1.12	1.03
Sleep disturbances	1.33	1.05
Use of sleeping medication	0.36	0.74
Daytime dysfunction	1.21	1.04
Total PSQI	7.54	5.39

Abbreviations: PSQI: Pittsburgh Sleep Quality Index; Sd: standard deviation.

**Table 3:** Univariate and multivariate logistic regression analyses for factors associated with SD in patients receiving ICIs.

Variable	Univariate OR (95% CI)	*p*	Multivariate OR (95% CI)	*p*
Female sex	1.84 (0.95–3.55)	0.070	1.44 (0.70–2.98)	0.316
Age ≥ 60 years	1.71 (0.96–3.05)	0.069	0.81 (0.39–1.65)	0.569
ECOG ≥ 1	3.29 (1.77–6.08)	<0.001	2.33 (1.17–4.62)	0.015
Second-line or beyond therapy	3.76 (1.92–7.34)	<0.001	2.43 (1.14–5.16)	0.021
≥2 metastatic sites	2.69 (1.47–4.92)	0.001	2.10 (1.06–4.16)	0.032
Number of ICI cycles ≥ 6	1.85 (1.04–3.32)	0.036	1.64 (0.86–3.13)	0.133

Abbreviations: OR: odds ratio; CI: confidence interval; ECOG: Eastern Cooperative Oncology Group.

## Data Availability

The data presented in this study are available on request from the corresponding author due to privacy and ethical restrictions related to patient confidentiality.

## Abbreviations

The following abbreviations are used in this manuscript:

CI	Confidence interval
CTLA-4	Cytotoxic T-lymphocyte-associated protein 4
CVD	Cardiovascular disease
DM	Diabetes mellitus
ECOG	Eastern Cooperative Oncology Group
HT	Hypertension
ICI	Immune checkpoint inhibitor
IQR	Interquartile range
IL	Interleukin
irAE	Immune-related adverse event
NSCLC	Non-small cell lung cancer
OR	Odds ratio
PD-1	Programmed cell death protein 1
PD-L1	Programmed death-ligand 1
PSQI	Pittsburgh Sleep Quality Index
RCC	Renal cell carcinoma
SD	Sleep disturbances
Sd	Standard deviation
TNF-α	tumor necrosis factor-alpha
